# Ulnar carpal translation following palmar locking plate fixation for distal radius fractures: a retrospective analysis

**DOI:** 10.1186/s13018-024-04689-w

**Published:** 2024-04-04

**Authors:** Shan-Yang Huang, Hui-Kuang Huang, Chin-Hsien Wu, Chih-Hsun Chang, Yi-Chao Huang, Jung-Pan Wang

**Affiliations:** 1 Department of Orthopedics, Ditmanson Medical Foundation Chiayi Christian Hospital, Chiayi, Taiwan; 2 Department of Orthopedics & Traumatology, Taipei Veterans General Hospital, Taipei, Taiwan; 3 Department of Surgery, School of Medicine, National Yang Ming Chiao Tung University, Taipei, Taiwan; 4 Department of Food Nutrition, Chung Hwa University of Medical Technology, Tainan, Taiwan; 5 Department of Orthopedics, E-Da Hospital, I-Shou University, Kaohsiung, Taiwan; 6 School of Medicine, College of Medicine, I-Shou University, Kaohsiung, Taiwan; 7 Department of Orthopedics, National Cheng Kung University Hospital, Tainan, Taiwan; 8 College of Medicine, National Cheng Kung University, Tainan, Taiwan

**Keywords:** Carpal instability, Distal radial fracture, Rotatory palmar subluxation of the lunate, Ulnar translation, Ulnar translocation

## Abstract

**Background:**

Concomitant injuries to the radiocarpal ligaments may occur during episodes of distal radius fractures, which may not cause acute subluxation or dislocation but can lead to radiocarpal instability and progress over time. This study aimed to analyze the occurrence of ulnar carpal translation (UCT) after open reduction and internal fixation of distal radius fractures and evaluate the associated factors of UCT.

**Methods:**

The retrospective study has been done now and includes patients treated between 2010 and 2020 who had undergone reduction and locking plate fixation of distal radius fractures. We assessed radiographs taken immediately after the operation and at 3 months post-operation, enrolling patients with UCT for evaluation. In addition to demographic data, we evaluated radiographic parameters, including fracture pattern, fragment involvement, and ulnar variance. We also assessed the palmar tilt-lunate (PTL) angle to determine associated rotatory palmar subluxation of the lunate (RPSL).

**Results:**

Among the 1,086 wrists, 53 (4.9%) had UCT within 3 months post-operation. The majority of wrists with UCT exhibited normal to minus ulnar variance (49 wrists; mean: −1.1 mm), and 24 patients (45.3%) had concomitant RPSL. Fracture classification was as follows: 19 type A3 (35.8%), 5 type C1 (9.4%), 11 type C2 (20.8%), and 18 type C3 (34.0%). Radial styloid was involved in 20 wrists (37.7%), palmar rim in 18 wrists (34.0%), dorsal rim in 25 wrists (47.2%), and die-punch fractures in 3 wrists (5.7%). Concomitant ulnar styloid fractures were present in 29 wrists (54.7%).

**Conclusion:**

This study highlights the potential for UCT to occur following reduction and fixation of distal radius fractures, particularly in cases with a more severe fracture pattern and combined with ulnar minus variance. The high incidence of concomitant RPSL provides further evidence for the possibility of associated radiocarpal ligament insufficiency after distal radius fracture.

## Background

Ulnar carpal translation (UCT) is a sequelae that mainly results from wrist ligament injury or attenuation, commonly seen in rheumatoid arthritis [1]. Post-traumatic UCT, first described by Linscheid et al. in 1972, is relatively uncommon [2, 3]. The radioscaphocapitate and palmar radiolunate ligaments play a key role in preventing UCT. Injuries to these ligaments can occur in various wrist injury mechanisms, and multiple ligaments may be involved [4–6]. There are various force and injury mechanisms that can lead to distal radius fractures, and UCT may occur as a result of ligament injuries sustained during episodes of distal radius fractures [7].

The Dumontier classification is commonly used to describe radiocarpal dislocation, where group 1 injuries are purely ligamentous, and group 2 injuries involve bony fractures in the radius with the ligaments attached intact to the fractured fragments [8]. However, studies have shown that patients with group 2 injuries may still experience residual radiocarpal instability and UCT even after open reduction and internal fixation of the fractured fragments [9]. In cases where a distal radius fracture presents itself, which has undergone a similar mechanism that could possibly lead to radiocarpal dislocation but has resulted in a distal radius fracture without dislocation appearance, it is likely that this type of fracture involves concomitant injury to the radiocarpal ligaments, similar to a group 2 injury.

Concomitant ligament injuries may occur in cases of distal radius fractures, but they may not always be severe enough to cause acute instability. Instead, resulting sequela related to these injuries may progress over time. Given the rarity of reports on UCT after distal radius fractures and the various injury mechanisms involved in the distal radius fractures, our study aims to answer the following questions: (1) Can UCT occur after open reduction and internal fixation of distal radius fractures? (2) What factors are associated with UCT following fixation of distal radius fractures?

## Materials and methods

We performed a retrospective evaluation of patients who underwent surgical fixation for distal radius fractures using a palmar approach with locking plate fixation between 2010 and 2020. The study design was approved by the Institutional Review Board of our institute. Informed consent was waived due to the retrospective nature of this study.

We included patients who underwent surgery within 2 weeks after the fracture episode to ensure uniformity in the timing of surgical intervention. We enrolled patients who had wrist radiographs taken in two views at postoperative 1 week and 3 months to ensure consistent radiographic evaluation during these time periods. As most patients were allowed to return to work and activities after 3 months, the influence of force loading would become significant beyond this point.

The enrolled patients must have undergone adequate reduction of their distal radius fractures, as defined by radial shortening < 5 mm, radial inclination > 15°, dorsal tilting < 15°, and articular step-off < 2 mm [10].

We excluded the following patients from our study: (1) patients with associated scapholunate (SL) or lunotriquetral (LT) injury, as evidenced by SL or LT mid-interval distance of more than 2.5 mm or an LT step-off [11, 12]; (2) patients aged younger than 20 years; (3) patients with previous wrist fracture or dislocation on the involved side; (4) patients with neurological injury or deficit in the involved limb; (5) patients presenting radiocarpal dislocation, including Dumontier types I and II [8]; and (6) patients with immunological disease or renal failure undergoing dialysis.

**Fig. 1:**
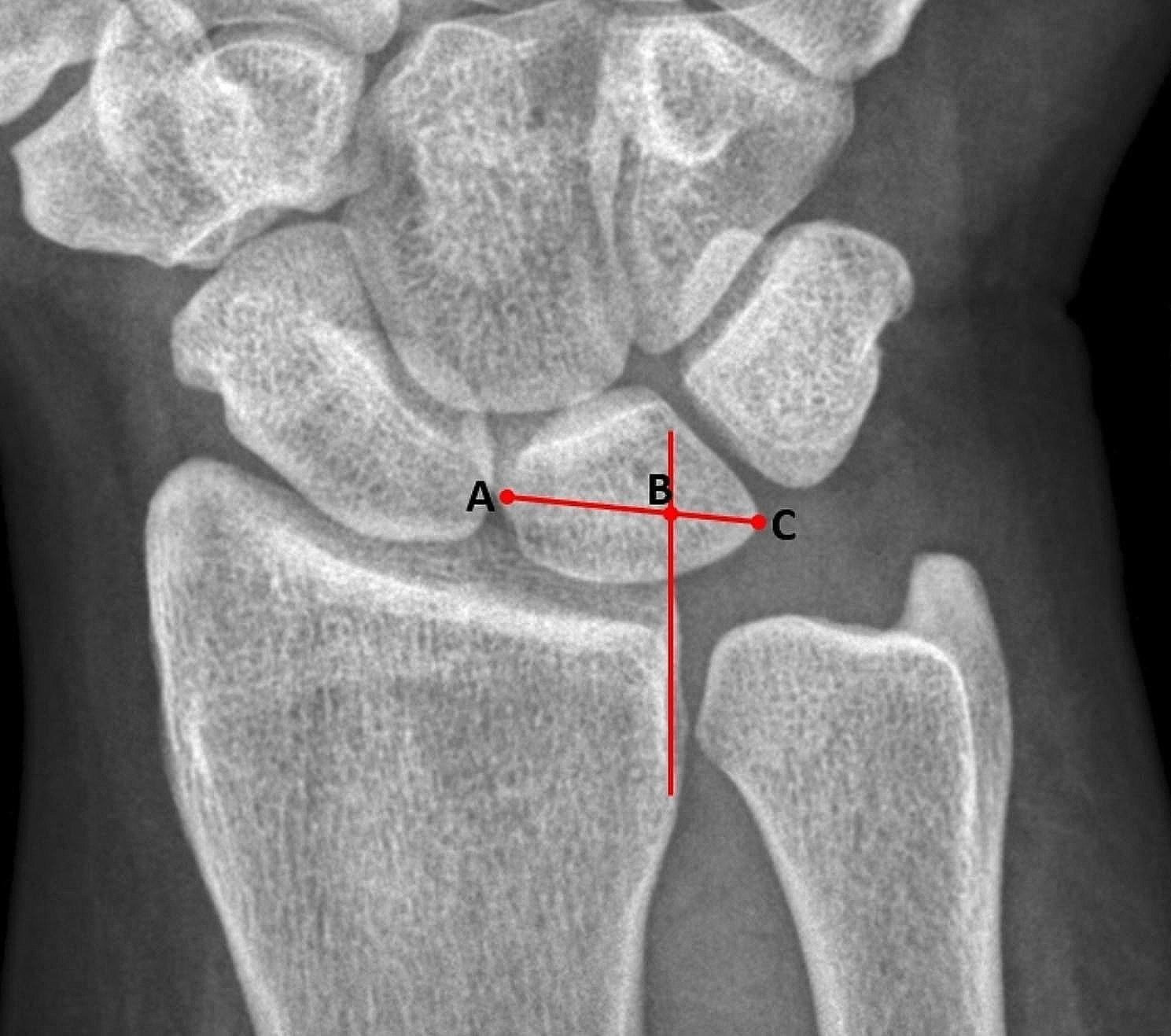
Measurements of the Gilula method for ulnar carpal translation (UCT) were taken from the farthest point on the radial border of the lunate (point **A**) to the farthest ulnar point at the mid-portion of the lunate (point **C**). A line drawn along the ulnar border of the radius, parallel to the long axis of the radius, intersects line AC at point **B**. The distance of the uncovered lunate (from point **B** to **C**) was divided by the full width of the lunate (from point **A** to **C**) and expressed as a percentage. A value greater than 44% indicates the presence of UCT

To assess UCT, we used the Gilula method, which involves measuring the degree of overhang of the lunate beyond the distal radial lunate facet. A value greater than 44% indicates the presence of UCT (Fig. 1) [13, 14].

In addition to evaluating demographic data and radiographic fracture patterns, we measured the palmar tilt-lunate (PTL) angle from lateral radiographs. This angle is determined by drawing lines that are tangential to the dorsal and palmar rims of the distal radius and the horns of the lunate, and a third line that is perpendicular to the lunate horn line and intersects with the radial-rim line. An angle greater than 110 degrees indicates rotatory palmar subluxation of the lunate (RPSL) [3, 15].

The interobserver reliability and enrollment for the study were established by two independent hand surgeons, each with more than 5 years of clinical practice in hand surgery. Both observers confirmed the presence of UCT and RPSL. To ensure the accuracy of the results, the intraobserver reliability was evaluated by asking the two observers to repeat the assessment after a one-month interval, thus minimizing any potential recall bias. This process involved confirming the presence of UCT or RPSL twice to ensure consistency. The fracture pattern of the distal radius, as well as any associated ulnar or carpal injuries, was confirmed and agreed upon by two other authors.

The data obtained for the study were analyzed using IBM SPSS Statistics version 18. The Chi-square test was used to compare differences in the discrete variable between the UCT and non-UCT groups. The significance level was set at *p* < 0.05.

## Results

Initially, we identified 2,218 wrists which had undergone surgery for distal radius fractures for evaluation. After applying our inclusion and exclusion criteria, we selected 1,086 wrists (1052 patients) that underwent reduction and locking plate fixation using a palmar approach, and met the criteria for adequate fracture reduction. These wrists were included in the radiographic evaluation.

**Fig. 2:**
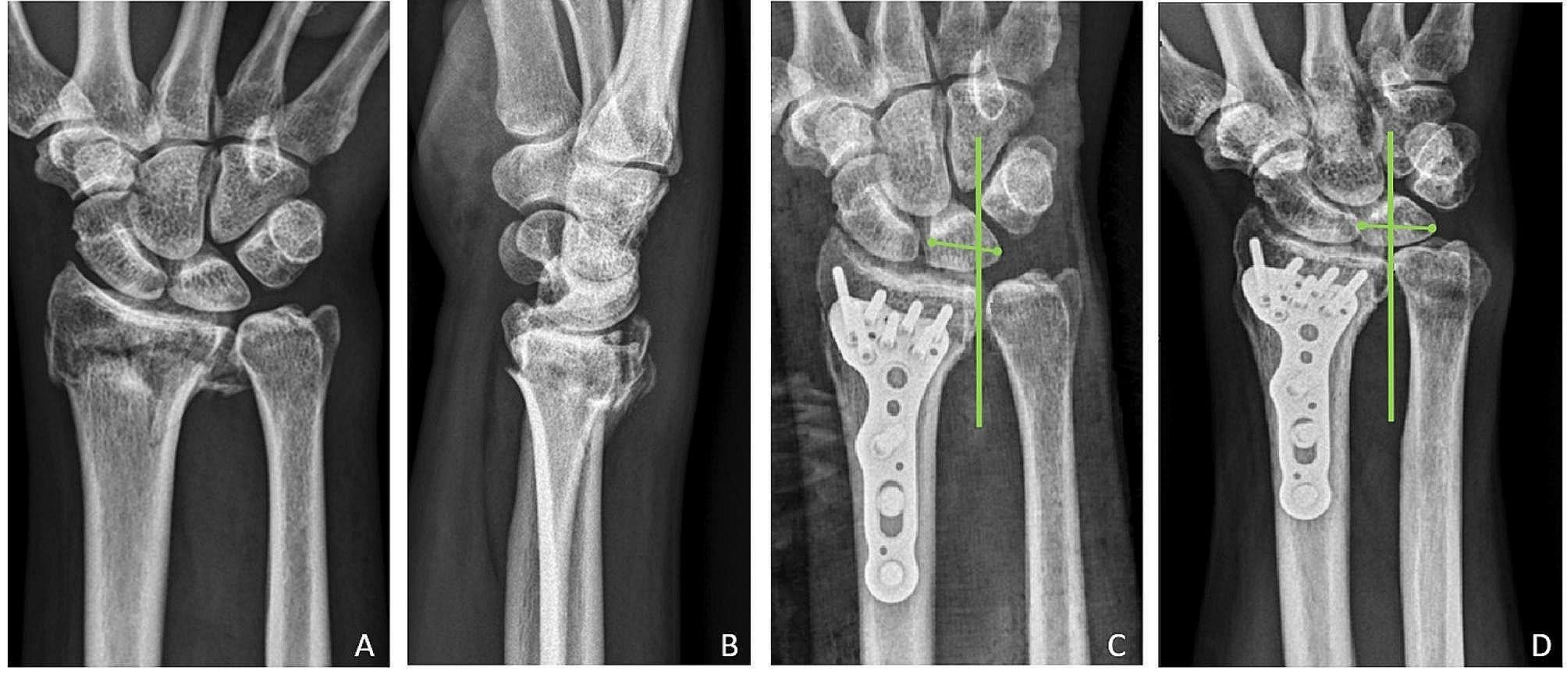
A 61-year-old man with right distal radius fracture. Radiographs showing (**A**) and (**B**) the fracture before treatment. (**C**) Radiograph after open reduction and internal fixation. (**D**) Ulnar carpal translation (UCT) was observed in the 3-month postoperative radiographs

**Fig. 3:**
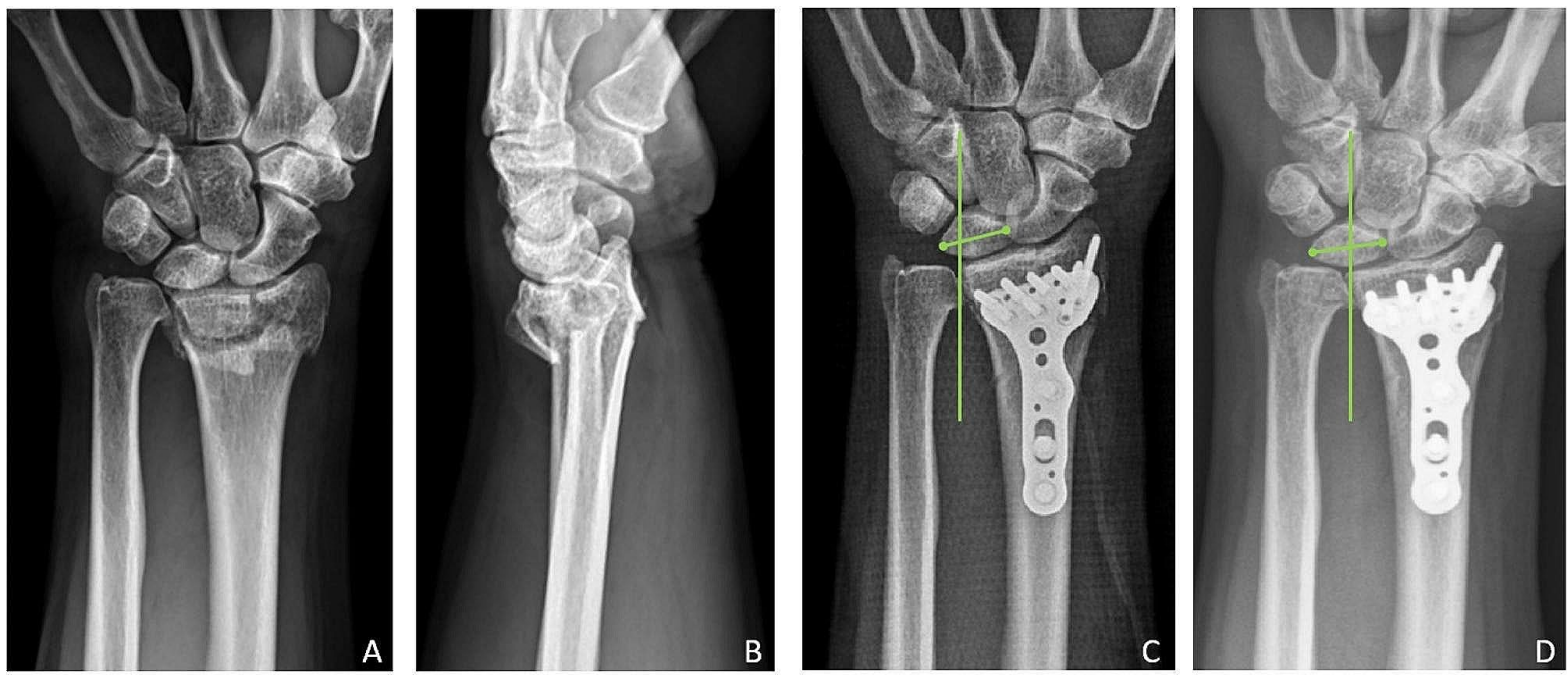
A 64-year-old man with left distal radius fracture. Radiographs showing (**A**) and (**B**) the fracture before treatment. (**C**) Radiograph after open reduction and internal fixation. (**D**) Ulnar carpal translation (UCT) was observed in the 3-month postoperative radiographs

A total of 53 wrists (4.9% of 1086 wrists) in 53 patients were noted to have UCT within 3 months after undergoing palmar plating for their distal radius fractures (Figs. 2 and 3). There were 15 male and 38 female with the mean age of these patients was 56.2 years (range: 20–88; SD: 16.1), and the mean BMI (body mass index) was 24.0 (range: 16.0–34.2; SD: 4.1). Based on the AO classification, these fractures consisted of 19 type A3 (35.8%), 5 type C1 (9.4%), 11 type C2 (20.8%), and 18 type C3 (34.0%)  . The Type A3, C2, and C3 fractures statistically appear more frequently in the UCT group compared to the non-UCT group (Table 1 and 2).

**Table 1:** Distribution of AO fracture classifications among patients with and without ulnar carpal translation (UCT)

AO classification	UCT (53 wrists)	Non–UCT (1033 wrists)	P value^a^
A1	0	0	–
A2	0	672 (65.1%)	< 0.001
A3	19 (35.8%)	20 (1.9%)	< 0.001
B1	0	6 (0.6%)	1.000
B2	0	27 (2.6%)	0.395
B3	0	160 (15.5%)	0.003
C1	5 (9.4%)	93 (9.0%)	1.000
C2	11 (20.8%)	30 (2.9%)	< 0.001
C3	18 (34.0%)	25 (2.4%)	< 0.001

^a^Chi-square test

Fracture alignment was reduced by an average of 8.6 degrees (range: 0–16 degrees; SD: 4.6 degrees) for palmar tilting and 21.9 degrees (range: 15–27 degrees; SD: 3.2 degrees) for radial inclination. In the review of our 53 enrolled patients, we noted that none of them underwent the intraarticular window-extended approach for fracture reduction, which would have reduced bias related to the volar plating soft tissue approach, as it can violate the integrity of the volar rim ligaments as part of the extended approach.

**Table 2:** Age and AO fracture classification distribution of patients with ulnar carpal translation (UCT)

Age (yr)	AO classification				Number (%)
	A3	C1	C2	C3	
20–29	3	0	0	2	5 (9.4%)
30–39	2	0	0	0	2 (3.8%)
40–49	3	1	3	2	9 (17.0%)
50–59	2	3	4	2	11 (20.8%)
60–69	6	1	4	7	18 (34.0%)
70–79	1	0	0	4	5 (9.4%)
80–89	2	0	0	1	3 (5.7%)
Total	19	5	11	18	53

Regarding fracture involvement, the radial styloid was involved in 20 wrists (37.7%), the palmar rim in 18 wrists (34.0%), the dorsal rim in 25 wrists (47.2%), and die-punch fractures in 3 wrists (5.7%). The involvement of the radial styloid, palmar rim, and dorsal rim is significantly more frequent in the UCT group compared to the non-UCT group. Concomitant ulnar styloid fractures were present in 29 wrists (54.7%), significantly more frequently than in the non-UCT group (320 wrists, 31.0%) (Table 3). Additionally, 24 wrists (45.3%) in the UCT group presented a significantly higher incidence of RPSL compared to 162 wrists (15.7%) in the non-UCT group, as the fracture involvement associated with RPSL in the UCT group was listed in Table 4.

**Table 3:** Involvement of fracture location, positive ulnar variance, and presence of rotatory palmar subluxation of the lunate (RPSL) in wrists with and without ulnar carpal translation (UCT)

Fracture involvement	UCT (53 wrists)	Non-UCT (1033 wrists)	P value^a^
Radial styloid	20 (37.7%)	47 (4.5%)	< 0.001
Palmar rim	18 (34.4%)	179 (17.3%)	0.004
Dorsal rim	25 (47.2%)	252 (24.4%)	< 0.001
Die-punch fracture	3 (5.7%)	29 (2.8%)	0.399
Ulnar styloid fracture	29 (54.7%)	320 (31.0%)	< 0.001
Positive ulnar variance	4 (7.5%)	260 (25.2%)	0.005
RPSL	24 (45.3%)	162 (15.7%)	< 0.001

^a^Chi-square test

**Table 4:** Distribution of fracture location involvement for the wrists with ulnar carpal translation (UCT) with or without rotatory palmar subluxation of the lunate (RPSL)

Fracture involvement	UCT with RPSL (24 wrists)	UCT without RPSL (29 wrists)	Total
Radial styloid	10 (50.0%)	10 (50.0%)	20
Palmar rim	12 (66.7%)	6 (33.3%)	18
Dorsal rim	14 (56.0%)	11 (44.0%)	25
Die-punch fracture	2 (66.7%)	1 (33.3%)	3
Ulnar styloid fracture	10 (34.5%)	19 (65.5%)	29

Among these UCT wrists, the majority had a normal to negative ulnar variance with a mean of −1.1 mm (range: 0– −3.5 mm; SD: 1.0 mm), while only 4 wrists (7.5%) had a positive ulnar variance. As for the non-UCT group, there were significantly more wrists with a positive ulnar variance, totaling 260 wrists (25.2%) (Table 3).

## Discussion

This study highlights that distal radius fractures can result not only in bony injuries but also in possible ligamentous injuries. One often overlooked complication is injury to the radiocarpal ligaments, which can lead to UCT and be associated with RPSL. Analysis of the fracture patterns revealed that greater fracture severity and the presence of associated ulnar styloid fractures were more likely to be associated with UCT. Furthermore, UCT was predominantly found in cases with ulnar neutral to minus variance.

It was reported that the radioscaphocapitate and radiolunate ligaments are primarily responsible for a restraint againt UCT [4, 16, 17]. Viegas et al. also reported that transection of the radioscaphocapitate and palmar radiolunate ligaments only results in palmar subluxation of the scaphoid and lunate during the cadaveric loading test. When combined with transection of the dorsal radiolunotriquetral ligament, slight ulnar shift of the carpus can occur [5]. More complete dissection of both sides of the palmar and dorsal radiocarpal ligaments could cause a substaintial UCT [6, 18]. Therefore, the more complex the combination of multiple radiocarpal ligament injuries, the more likely it is to result in UCT [5, 19]. According to our findings, we observed that patients with UCT tended to have more severe fracture patterns, such as 35.8% AO type A3, 20.8% C2, and 34.0% C3 fractures, along with a concomitant ulnar styloid fracture in 54.7% of cases, while the non-UCT group exhibited significantly fewer occurrences of A3 (1.9%), C2 (2.9%), and C3 (2.4%) fractures, as well as fewer accompanying ulnar styloid fractures (31.0%). These severe fracture patterns may suggest the existence of multiple ligament injuries.

The majority of wrists with UCT exhibited normal to minus ulnar variance (49 wrists; mean: −1.1 mm), with positive ulnar variance significantly more prevalent in 4 out of 53 (7.5%) UCT wrists compared to 260 out of 1033 (25.2%) non-UCT wrists. These findings are consistent with previous studies by Rayhack et al. and Berschback et al., which also reported that traumatic UCT cases were mainly presented with neutral to minus ulnar variance [3, 4]. Hsu et al. reported their series that all patients who developed further UCT during follow-up after open reduction and internal fixation of distal radius fractures in Dumontier group 2 cases had ulnar minus variance [9].

It is unclear whether a lower percentage of loading from the radius in ulnar positive variance would provide an advantage in avoiding UCT in patients with radiocarpal ligament insufficiency [20], or if the hypothesis that a loss of ulnar buttressing through the ulnar head and triangular fibrocartilage may play a role in the pathomechanics of UCT [4, 21, 22]. However, it is important to note that patients with more severe distal radius fracture patterns combined with ulnar minus variance after fracture reduction and internal fixation may have a higher risk of developing UCT.

**Fig. 4:**
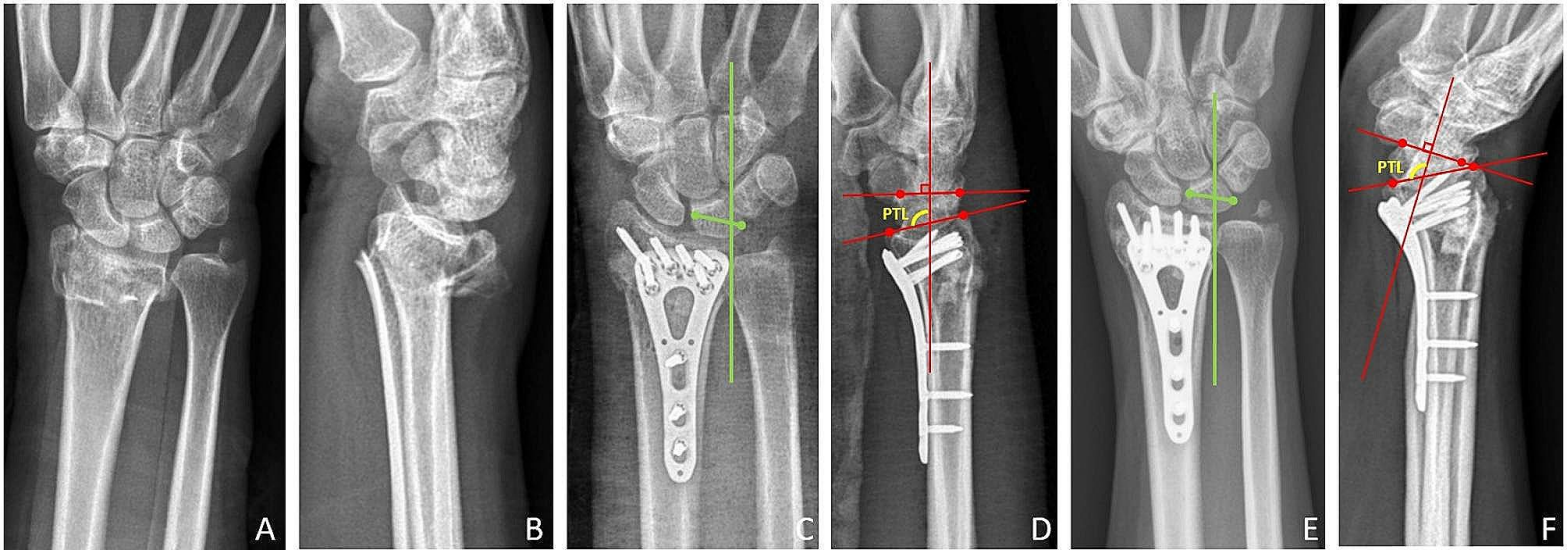
A 63-year-old woman with right distal radius fracture. Radiographs showing (**A**) and (**B**) the fracture before treatment; (**C**) and (**D**) after open reduction and internal fixation. (**E**) and (**F**) Radiographs taken 3 months postoperatively, revealing ulnar carpal translation (UCT) and rotatory palmar subluxation of the lunate (RPSL) as evidenced by an increase in the palmar tilt-lunate (PTL) angle

According to Stäbler et al., UCT is typically associated with RPSL, which is characterized by an increase in the PTL angle (Fig. 4). This phenomenon is often caused by injury to the palmar-radial ligaments of the wrist, which work against the tendency of the lunate to rotate dorsally and subluxate palmarly. Therefore, injuries to the palmar radial ligaments are significant causes of both UCT and RPSL.

The association between UCT and palmar or rotatory palmar subluxation of the carpus has been recognized as a cause of attenuation of the palmar radial ligaments [3, 5, 15, 19, 23]. Our study also found a high percentage (45.3%) of UCT cases combined with the appearance of RPSL compared to 162 wrists (15.7%) in the non-UCT group, which tends to be consistent with this association.

In addition, among the 18 patients (34.4%) with fractures involving the palmar rim (while the non-UCT group presented significantly less palmar rim involvement of 17.3%), 12 had concomitant RPSL. This number is twice the number of the 6 wrists without RPSL.This finding suggests that if fractures involve the palmar rim, it is more likely to result in palmar ligament injuries and insufficiency, which can possibly lead to the development of UCT combined with RPSL.

In this study, 29 wrists (54.7%) with UCT were associated with ulnar styloid fractures. Interestingly, 19 of these wrists did not present with RPSL, which was almost twice the number of wrists with RPSL injuries. It is worth noting that the presence of UCT is more often associated with fractures involving both the distal radius and ulnar styloid. However, it is still unclear whether the force or mechanism of injury is strong enough to cause radiocarpal instability and fractures on both sides of the wrist. When the force loading is transmitted towards or shared by the ulnar side, it may result in less radiocarpal instability, reducing the likelihood of RPSL.

In our study of UCT patients, we observed a relatively low incidence of die-punch fractures, with only three cases (5.7%) reported, which is also consistent with no difference in the non-UCT group with 29 wrists (2.8%). The Fernandez classification categorizes distal radius fractures into five distinct injury mechanisms, and it identifies die-punch fractures as mainly associated with compression forces [7]. This mechanism is less likely to cause damage to the key ligaments responsible for maintaining radiocarpal stability, as compared to other mechanisms such as bending, shearing, or avulsion.

Surgical treatment with ligament repair or reinforcement for the UCT may not be able to fully restore the normal radiocarpal relationship, but it can help prevent the worsening of ulnar translation of the carpus [3, 4, 9, 18, 24]. Our study identified some factors that may associate with the UCT following distal radius fractures. Although we could not provide sufficient data to support the efficacy of these concomitant treatments for radiocarpal instability, addressing the issue during distal radius osteosynthesis surgery is the optimal timing. When the palmar ligaments were repaired late in UCT cases, the outcomes were often unsatisfactory; as a result, partial radiocarpal arthrodesis is frequently recommended [3, 25, 26].

Rayhack et al. reported eight cases of posttraumatic UCT with an average delayed diagnosis of 7.3 months (range 2–23 months) [4]. In contrast, our study evaluated cases only up to three months after the injury due to the heterogeneous follow-up period of all the reviewed patients. Longer follow-up periods could potentially reveal more cases of UCT, but many patients do not return for follow-up after three months of surgery. We believe that UCT following distal radius osteosynthesis could potentially impair wrist function or lead to incomplete wrist function recovery. In presenting the findings of this study, we would like to caution against the possible problem of radiocarpal instability combined with distal radius fractures and encourage further studies to improve our understanding and management of this issue.

Our study had several limitations, primarily due to its retrospective nature. The short-term radiographic evaluation precluded our ability to evaluate cases with delayed presentation or assess further sequelae such as osteoarthritis. The involvement of multiple surgeons may have led to bias in our results. When evaluating UCT, bias can arise from radiographs and measurements. We employed the Gilula method, which defines UCT as when more than 44% of the lunate extends beyond the distal radial lunate facet. To minimize bias, we focused solely on identifying the presence of this phenomenon using a single cutoff point and did not utilize percentage values of the overhanging lunate for evaluating other data correlations. Additionally, our review lacked complete functional outcomes data, which prevented us from quantifying the clinical impact of the UCT.

## Conclusion

Based on this study, we presented a rare phenomenon of UCT that may gradually develop after the fixation of distal radius fractures, especially in patients severe distal radius fracture patterns and ulnar minus variance. The high incidence of concomitant RPSL provides further evidence for the possibility of associated radiocarpal ligament insufficiency after distal radius fractures. Further research is needed to better understand this traumatic phenomenon, its contributing factors, and functional impact, and to develop effective strategies for prevention and treatment.

## Data Availability

No datasets were generated or analysed during the current study.
